# Earlier Prostatic Artery Embolization for Progressive Benign Prostatic Hyperplasia: A Biological Rationale and Hypothesis for Disease Interception

**DOI:** 10.7759/cureus.116496

**Published:** 2026-09-18

**Authors:** Julian Sadowski

**Affiliations:** 1 Medicine, Independent Research, Ocala, USA

**Keywords:** acute urinary retention, benign prostatic hyperplasia, bladder outlet obstruction, bladder remodeling, detrusor underactivity, disease progression, lower urinary tract symptoms, prostatic artery embolization

## Abstract

Benign prostatic hyperplasia (BPH) is a heterogeneous progressive disease that can culminate in bladder outlet obstruction, acute urinary retention (AUR), and potentially irreversible bladder dysfunction. Current treatment pathways appropriately prioritize symptom burden, patient preference, and established complications; however, symptom severity correlates imperfectly with obstruction and detrusor reserve. Prostatic artery embolization (PAE) improves lower urinary tract symptoms with a lower immediate procedural and sexual adverse-event burden than transurethral surgery but produces less complete objective de-obstruction and a greater need for retreatment. These tradeoffs raise a distinct question: whether PAE should be studied earlier in carefully selected patients with objective evidence of progression before AUR or advanced detrusor decompensation occurs. This narrative review critically examines the natural history of BPH, bladder remodeling and its uncertain reversibility, limitations of symptom-based risk assessment, and comparative evidence for PAE, medical therapy, transurethral resection of the prostate, and holmium laser enucleation of the prostate. The evidence establishes that PAE is effective for symptomatic disease and can improve selected urodynamic measures; it does not establish that PAE prevents AUR, arrests bladder remodeling, or preserves long-term detrusor function. No validated marker currently identifies an impending point of irreversible decompensation, and earlier intervention could expose patients with stable disease to contrast, radiation, vascular complications, technical failure, and retreatment. The available evidence therefore supports clinical equipoise rather than a practice recommendation. A randomized trial using progression and bladder-function endpoints is needed to determine whether a reproducibly defined high-risk phenotype benefits from earlier PAE compared with optimized medical management.

## Introduction and background

Benign prostatic hyperplasia (BPH) is one of the most prevalent chronic diseases affecting aging men and the leading cause of lower urinary tract symptoms (LUTS) worldwide [1]. Although many patients experience mild symptoms that remain stable for years, others demonstrate progressive bladder outlet obstruction despite medical therapy, resulting in worsening urinary symptoms, acute urinary retention (AUR), recurrent catheterization, and declining quality of life. Current management strategies appropriately emphasize conservative management with lifestyle modification, medical therapy, and watchful waiting for patients without complications. However, these approaches are largely guided by symptom severity rather than objective markers of disease progression, raising the possibility that clinically significant bladder remodeling may occur before intervention is considered.

BPH is increasingly recognized as a progressive disease rather than a static enlargement of the prostate. Persistent bladder outlet obstruction may be associated with compensatory detrusor hypertrophy, increased voiding pressure, extracellular matrix remodeling, impaired contractility, and chronic retention [2]. The sequence, timing, and reversibility of these changes are heterogeneous, and functional recovery after de-obstruction varies among patients. These observations raise the possibility that the timing of intervention may influence long-term bladder outcomes. 

AUR represents one of the most clinically significant manifestations of disease progression. Traditionally regarded as a complication requiring urgent decompression, AUR may, in some patients, occur in the setting of advanced outlet obstruction or diminished detrusor reserve, although it is not a reliable surrogate for irreversible bladder dysfunction. Patients presenting with AUR or advanced urinary retention may exhibit large residual volumes and impaired detrusor contractility, and functional recovery after subsequent outlet surgery can be variable [3,4]. Although these findings do not establish causality, they raise an important clinical question: can patients at highest risk for progression be identified before clinically significant bladder dysfunction develops? 

Prostatic artery embolization (PAE) has emerged as a minimally invasive treatment for symptomatic BPH that improves LUTS, urinary flow, and quality of life while avoiding many of the perioperative risks and sexual adverse effects associated with transurethral surgery [5,6]. Because of its favorable safety profile and lower procedural burden, PAE introduces a therapeutic option that may permit consideration of earlier intervention in carefully selected patients who demonstrate objective evidence of progressive disease despite optimized medical therapy. Whether intervention before AUR or advanced bladder decompensation can preserve bladder function remains unknown and has not been adequately studied.

This narrative review examines the biological rationale linking progressive bladder outlet obstruction to bladder dysfunction, reviews the evidence surrounding delayed intervention and clinical outcomes, and proposes the hypothesis that earlier PAE may merit prospective investigation as a strategy to alter disease progression in carefully selected patients with progressive BPH. Rather than advocating a change in current clinical practice, this review identifies an important knowledge gap and outlines a framework for future studies evaluating whether earlier minimally invasive de-obstruction can preserve bladder function and reduce progression to AUR.

## Review

Literature review approach

This article was developed as a narrative review rather than a systematic review. Targeted searches of PubMed and reference lists of relevant guidelines, randomized trials, systematic reviews, and large observational studies were conducted through August 23, 2026. Search concepts included BPH, benign prostatic obstruction, LUTS, AUR, post-void residual volume, detrusor underactivity, bladder remodeling, de-obstruction, PAE, transurethral resection of the prostate (TURP), holmium laser enucleation, medical therapy, retreatment, and urodynamics. Studies were selected for inclusion based on direct relevance to the proposed disease-interception hypothesis, with particular emphasis on evidence addressing the natural history of BPH, progression to AUR or bladder dysfunction, objective measures of obstruction, comparative treatment effectiveness, and durability of outcomes.

Priority was given to professional guidelines, randomized comparative trials, systematic reviews, prospective studies, and large cohorts that addressed disease progression, objective obstruction, bladder function, AUR, durability, or treatment-related harm. Seminal older studies were retained when they defined natural history, biological mechanisms, or commonly used outcome measures. Reports focused solely on technical variants without clinically relevant outcomes were not emphasized.

Because the purpose was hypothesis development and critical synthesis, study selection was not performed with a prespecified systematic-review protocol, formal risk-of-bias instrument, or meta-analysis. The conclusions should therefore be interpreted as a structured appraisal of the evidence and its gaps, not as a quantitative estimate of comparative treatment effect.

Progressive bladder outlet obstruction and bladder remodeling in benign prostatic hyperplasia

BPH is characterized by progressive proliferation of prostatic stromal and epithelial tissue that increases prostate volume and smooth muscle tone, producing bladder outlet obstruction of varying severity. Although disease progression is heterogeneous, many patients experience worsening obstruction over time despite medical therapy. Chronic outlet obstruction contributes not only to LUTS but also to recurrent urinary tract infections, bladder calculi, hematuria, renal dysfunction, and AUR, underscoring that BPH is a progressive disorder rather than a static condition [1,7].

Natural history and clinical risk stratification

Progression is clinically heterogeneous. Some men remain stable during observation, whereas others experience symptom deterioration, reduced urinary flow, recurrent infection, bladder stones, renal consequences, AUR, or a need for surgery. Long-term trials established that prostate volume and prostate-specific antigen, used as a surrogate for gland size after excluding malignancy, are associated with progression risk, while symptom burden alone is a less specific predictor of AUR [3,8,9]. This distinction supports risk stratification but does not create a validated threshold for prophylactic intervention.

Medical therapy modifies this natural history. In medical therapy of prostatic symptoms (MTOPS), finasteride and combination therapy reduced overall clinical progression and the risks of AUR and invasive treatment, while doxazosin primarily improved symptoms [8]. Combination of Avodart and tamsulosin (CombAT) similarly showed that dutasteride plus tamsulosin reduced progression more effectively than either agent alone in men selected for increased progression risk [9]. These trials are a major argument against portraying current management as passive: contemporary medical therapy already functions as disease-modifying prevention for many men with enlarged prostates.

The remaining problem is not universal failure of medical management but residual risk. Progression may occur despite appropriate treatment, and real-world adherence is substantially lower than adherence in randomized trials [10]. Adverse sexual effects, orthostatic symptoms, perceived lack of benefit, polypharmacy, and preference against indefinite medication can reduce persistence [10,11]. Earlier PAE would therefore need to demonstrate benefit beyond optimized and adherent medical therapy, not merely outperform untreated observation.

A candidate disease-interception strategy must also distinguish an enlarged prostate from clinically important obstruction. Prostate volume, PSA, maximum urinary flow rate (Qmax), post-void residual volume (PVR), intravesical prostatic protrusion, bladder morphology, and pressure-flow studies describe different aspects of the disease. None alone proves progressive loss of detrusor reserve. A composite phenotype is biologically more plausible than any single cutoff, but it remains unvalidated.

Clinical manifestations and limitations of symptom-based assessment

LUTS are the principal clinical manifestation of BPH and become increasingly prevalent with age. Moderate-to-severe symptoms can impair sleep, daily functioning, psychological well-being, and overall quality of life [1]. However, symptom burden alone does not reliably indicate the severity of outlet obstruction or underlying bladder dysfunction. Storage, voiding, and post-micturition symptoms may arise from numerous conditions in addition to BPH, including detrusor overactivity, impaired bladder contractility, neurologic disease, diabetes mellitus, medication effects, and other lower urinary tract disorders. Furthermore, patients with similar symptom scores may demonstrate markedly different degrees of bladder remodeling, post-void residual volume, urinary flow impairment, and detrusor contractility. Consequently, reliance on symptoms alone may underestimate disease progression in some individuals while overestimating obstruction in others.

The International Prostate Symptom Score (IPSS) remains the most widely used and validated instrument for quantifying LUTS and monitoring treatment response. The questionnaire evaluates seven urinary symptoms encompassing storage and voiding domains in addition to a quality-of-life assessment, providing a standardized measure of symptom severity [12]. While IPSS is valuable for assessing patient-reported outcomes and guiding shared decision-making, it primarily reflects symptom burden rather than the structural or functional consequences of bladder outlet obstruction [2,7]. Objective measures may therefore provide complementary information when evaluating disease progression.

What objective markers can and cannot establish

Objective assessment can reveal discordance between symptoms and physiology, but each measure has limitations. A low Qmax may reflect outlet obstruction, reduced detrusor contractility, or an inadequately filled bladder. PVR varies between measurements and may increase because of obstruction, detrusor underactivity, medications, neurologic disease, or diabetes. Prostate enlargement increases population-level risk but does not establish that a particular patient is approaching decompensation. Structural findings such as trabeculation and diverticula suggest chronic pressure exposure but do not quantify reversibility [2,7,13].

Pressure-flow urodynamics remains the most direct method for distinguishing obstruction from impaired contractility, yet routine invasive testing is neither necessary nor practical for every patient with LUTS. Even urodynamic indices describe current function rather than reliably predicting the time at which remodeling will become irreversible. This is a central limitation of the proposed hypothesis: the biological therapeutic window is plausible, but it cannot presently be identified with a validated clinical rule.

Accordingly, established population-level predictors of BPH progression should be distinguished from proposed variables for trial enrichment. Prostate volume, PSA, age, symptom burden, and selected measures of urinary function have demonstrated associations with progression or retention risk, but rising PVR, declining Qmax, bladder morphology, and urodynamic findings do not individually or collectively define a validated threshold for impending detrusor decompensation. For a prospective trial, persistent changes confirmed on repeat testing could be evaluated alongside prostate anatomy, symptom evolution, medication response, comorbidity, and selective urodynamics as candidate enrichment variables. Their purpose would be to identify and validate a research phenotype with increased progression probability, not to serve as established indications for PAE.

Current management of progressive benign prostatic hyperplasia: strengths and limitations

Current management appropriately emphasizes shared decision-making and conservative care for men with mild or non-bothersome LUTS. Initial evaluation centers on clinical history, physical examination, symptom assessment, and urinalysis; PVR, uroflowmetry, and prostate size assessment may be used selectively and are particularly relevant when procedural treatment is considered [7]. This approach avoids unnecessary intervention for many men, but current guidelines do not define validated surveillance thresholds that identify impending detrusor decompensation.

The introduction of α-adrenergic antagonists and 5α-reductase inhibitors has transformed BPH into a chronic medically managed disease. Large randomized trials, including MTOPS and CombAT, demonstrated that combination therapy reduces symptom progression, decreases the risk of AUR, and lowers the need for subsequent surgical intervention [8,9]. These findings established medical therapy as the cornerstone of BPH management and significantly improved long-term outcomes for many patients.

Despite these advances, a clinically important proportion of patients experience continued symptom progression, urinary retention, or a need for procedural treatment. Long-term adherence also remains suboptimal, with population-based studies demonstrating that fewer than one-third of patients remain adherent after one year and fewer than one-fifth continue therapy after four years [10]. Furthermore, pharmacologic treatment may be associated with adverse effects including ejaculatory dysfunction, erectile dysfunction, and reduced libido, which may contribute to treatment discontinuation [11].

Importantly, current treatment algorithms remain largely symptom-driven. While periodic assessment of IPSS, urinary flow rate, and post-void residual volume is recommended, intervention is generally reserved for patients with bothersome symptoms or established complications. Consequently, patients with progressive bladder remodeling but relatively stable symptom scores may continue conservative management until bladder dysfunction has advanced. This raises an important unanswered question: can earlier identification of objective disease progression permit intervention before advanced bladder decompensation develops?

Management of acute urinary retention and the consequences of delayed intervention

AUR caused by benign prostatic obstruction requires immediate bladder decompression, most commonly through urethral catheterization and, when urethral access is unsuccessful or contraindicated, suprapubic catheterization. An α₁-adrenergic antagonist is generally initiated before a trial without catheter (TWOC) to improve the likelihood of spontaneous voiding. Contemporary practice typically favors a period of catheter drainage followed by TWOC rather than immediate surgical intervention, allowing the acute episode to resolve and, when necessary, permitting definitive treatment to be performed electively [7,14]. In the Reten-World survey, α₁-blocker administration before TWOC nearly doubled the likelihood of success, supporting its routine use in this setting [14].

Although TWOC can avoid prolonged catheterization and defer or eliminate the immediate need for surgery, its success is variable. Contemporary pooled evidence suggests that approximately one-half of patients regain spontaneous voiding after either immediate or delayed TWOC, although the available studies are heterogeneous and generally at substantial risk of bias [15]. In the Reten-World cohort, patients who failed an initial TWOC and underwent a second attempt achieved success in 29.5% of cases [14].

Several clinical features predict TWOC failure, including advanced age, severe baseline LUTS, prostate volume of at least 50 g, spontaneous rather than precipitated retention, and a drained bladder volume of at least 1,000 mL [14]. These factors are clinically important because they may identify patients in whom AUR occurs in association with more advanced outlet obstruction or diminished detrusor reserve, although they do not establish the degree or reversibility of underlying bladder dysfunction. In such patients, repeated TWOC attempts may restore voiding temporarily without correcting the underlying pathophysiology.

Prolonged catheterization also carries meaningful morbidity. In Reten-World, catheterization lasting more than three days was associated with higher rates of bacteriuria, urinary tract infection, urine leakage, catheter obstruction, and other catheter-related adverse events, although a longer catheter duration did not independently improve TWOC success [14]. Thus, the clinical objective is not simply to maximize the duration of bladder drainage, but to balance the possibility of recovery against the complications of continued catheter dependence.

Even after an initially successful TWOC, recurrent retention remains an important concern, particularly among older patients, those with large retained volumes, and those with evidence of impaired urinary flow or incomplete bladder emptying. Recurrent catheterization, emergency-department utilization, infection risk, and disruption of quality of life contribute substantially to the burden of AUR. Moreover, hospitalization with AUR is associated with increased subsequent mortality, although this relationship appears to be strongly influenced by patient age, frailty, and coexisting illness rather than establishing AUR itself as the direct cause of death [16].

These observations highlight an important limitation of the current treatment pathway. Catheterization and TWOC appropriately address the acute inability to void, but they do not directly reverse chronic bladder outlet obstruction or established detrusor remodeling. For some patients with high-risk features, AUR may occur in the setting of more advanced disease, but the event itself does not establish irreversible bladder dysfunction. By the time recurrent retention, catheter dependence, or TWOC failure occurs, the opportunity to preserve normal bladder function may already be diminishing. This provides a rationale for identifying progressive obstruction earlier and investigating whether selected patients could benefit from definitive deobstruction before AUR develops.

Progressive bladder remodeling and the transition to detrusor decompensation

Bladder outlet obstruction secondary to BPH initiates a dynamic process of structural and functional remodeling within the bladder. Rather than representing a static consequence of urethral obstruction, the bladder undergoes a continuum of adaptive and maladaptive changes in an attempt to maintain efficient urinary storage and emptying despite increasing outlet resistance. The severity and duration of obstruction, together with patient age and underlying bladder health, may influence whether compensatory mechanisms remain effective or are associated with progressive bladder dysfunction; however, the timing, sequence, and reversibility of these changes vary among patients [2,17].

During the early compensated phase, increasing outlet resistance stimulates detrusor smooth muscle hypertrophy and augmented contractility, allowing the bladder to generate higher voiding pressures and preserve urinary flow. Although these adaptations initially maintain bladder emptying, they are accompanied by progressive increases in intravesical pressure and mechanical stress. Elevated voiding pressures may produce repeated episodes of ischemia and reperfusion injury, triggering oxidative stress, chronic inflammation, extracellular matrix remodeling, and collagen deposition within the bladder wall [17,18]. Over time, these pathological changes reduce bladder compliance and elasticity while replacing functional smooth muscle with fibrotic connective tissue.

With sustained obstruction, compensatory changes may become maladaptive in some patients. Detrusor contractility declines, bladder sensation becomes impaired, and post-void residual urine progressively increases. Structural changes including bladder wall thickening, trabeculation, pseudodiverticula formation, and detrusor fibrosis may accompany progressive bladder dysfunction [2,13]. Importantly, these alterations often develop gradually over years and may not parallel symptom severity, resulting in patients who appear clinically stable despite progressive deterioration in bladder function. Objective measures such as increasing post-void residual volume, declining urinary flow rate, and changes in bladder wall morphology may therefore provide earlier evidence of disease progression than symptom scores alone.

AUR may occur within this heterogeneous course of progressive bladder outlet obstruction but should not be considered a uniform marker of advanced irreversible bladder dysfunction. AUR may, in some patients, represent a late manifestation of progressive outlet obstruction and diminishing detrusor reserve. Once detrusor underactivity and impaired bladder sensation become established, restoration of normal bladder function following relief of obstruction becomes increasingly unpredictable. Clinical studies demonstrate that some patients with detrusor underactivity recover meaningful voiding function after relief of obstruction, although outcomes remain heterogeneous [4,13].

Importantly, both experimental and clinical studies suggest that bladder remodeling exists on a spectrum of reversibility. Regression of detrusor hypertrophy and improvements in bladder wall thickness have been demonstrated following successful medical and surgical relief of obstruction, whereas recovery in the presence of more advanced fibrosis or detrusor underactivity appears more variable and may be incomplete [2,13]. Recent molecular studies further support the concept that relief of bladder outlet obstruction can normalize key pathways involved in bladder remodeling when intervention occurs before advanced decompensation develops [19]. Collectively, these findings raise the hypothesis that a therapeutic window may exist during which definitive treatment may preserve bladder function before advanced contractile impairment occurs. No validated clinical marker currently identifies such a therapeutic window; its existence and timing therefore remain hypothetical and require prospective investigation.

Why earlier prostatic artery embolization deserves investigation

PAE has emerged as a minimally invasive treatment for LUTS caused by BPH. By producing ischemic reduction of prostatic tissue, PAE decreases prostate volume and relieves the static component of bladder outlet obstruction without transurethral resection, general anesthesia, or surgical disruption of the bladder neck. Clinical studies have demonstrated improvements in symptom scores, quality of life, urinary flow, and post-void residual volume following PAE. Its lower perioperative burden and reduced risk of ejaculatory and other sexual adverse effects distinguish it from conventional transurethral surgery and may make it acceptable to patients who would otherwise defer procedural treatment [5,6].

Randomized evidence has also begun to examine PAE earlier in the BPH treatment pathway. Trials comparing PAE with combined medical therapy in treatment-naïve or medically managed patients have demonstrated greater improvements in urinary symptoms, quality of life, prostate volume, and measures of urinary obstruction after embolization [20,21]. These findings establish that PAE can provide clinically meaningful symptom improvement accompanied by measurable improvements in urinary flow, residual volume, and prostate size. They do not, however, demonstrate that PAE prevents AUR or preserves long-term detrusor function, because existing trials were designed primarily to evaluate symptom relief rather than disease progression.

Comparisons with TURP further define the potential role of PAE. TURP generally produces greater improvements in maximum urinary flow, post-void residual volume, and other objective measures of outlet obstruction. Five-year randomized data also indicate that the functional improvement and durability of PAE are inferior to those of TURP, with a greater likelihood of subsequent retreatment [5,6]. Conversely, PAE is associated with fewer perioperative complications, shorter recovery, and better preservation of ejaculatory function [5,6]. PAE should therefore not be presented as a universally equivalent substitute for TURP, particularly in patients requiring rapid or maximal relief of advanced obstruction.

These differences are central to the hypothesis proposed in this review. TURP remains an effective standard for established bladder outlet obstruction, urinary retention, and other BPH-related complications. However, its procedural burden and potential sexual adverse effects may discourage intervention while disease remains in an earlier compensated phase. PAE offers less complete de-obstruction but with a lower immediate treatment burden. This tradeoff raises the possibility that PAE could occupy a distinct role as an earlier intervention for patients showing objective progression despite medical therapy but who have not yet developed AUR, catheter dependence, or advanced detrusor dysfunction.

The proposed disease-interception strategy would not extend PAE to all patients with mild or stable LUTS. Appropriate candidates would need evidence suggesting continued progression, such as increasing post-void residual volume, declining maximum urinary flow, progressive prostate enlargement, worsening symptoms despite optimized medical therapy, or early structural or urodynamic evidence of bladder dysfunction. Patient selection would also require exclusion of alternative causes of LUTS and assessment of prostatic arterial anatomy, renal function, contrast risk, and the likelihood that PAE could be performed successfully.

At present, the preventive value of this approach remains hypothetical. Existing studies support the effectiveness and favorable safety profile of PAE for symptomatic BPH [5,6,20-22], but they do not establish that earlier embolization interrupts bladder remodeling or prevents AUR. Nevertheless, the combination of biological plausibility and the established symptomatic and functional efficacy of PAE creates clinical equipoise for prospective investigation of a separate, unproven question: whether earlier treatment can modify disease progression, preserve bladder function, or reduce AUR. The relevant question is therefore not whether PAE should replace TURP, but whether earlier PAE can improve the natural history of progressive BPH compared with optimized and adherent guideline-directed medical therapy.

Comparative Evidence: Prostatic Artery Embolization Versus Sham Treatment and Medical Therapy

The efficacy of PAE is not solely attributable to placebo response. In a sham-controlled trial of 80 men with severe, medically refractory LUTS, PAE produced substantially greater improvement in IPSS and quality of life at six months than sham embolization [23]. The study established a treatment effect but was single-center, included severe rather than early disease, and was not designed around AUR or detrusor preservation.

Prostatic artery embolisation versus medical treatment (PARTEM) compared PAE with combined medical therapy and demonstrated superior improvement in symptoms and quality of life after embolization in men with bothersome LUTS [20]. Prostate embolisation as first-line therapy compared to medication in treatment-naïve men with prostate enlargement, a randomised controlled trial (P-EASY ADVANCE) extended this comparison to treatment-naive men with enlarged prostates and urodynamically obstructed or equivocal findings; PAE improved symptoms, Qmax, incomplete emptying, prostate volume, and the proportion classified as unobstructed more than combination medication in a small randomized cohort [21]. Moreover, the relatively short follow-up of most available trials limits extrapolation of symptomatic and functional improvements to long-term disease-interception outcomes.

Recent urodynamic evidence adds mechanistic support while also illustrating uncertainty. In prostate artery embolisation safety and efficacy: preliminary and follow-up urodynamic studies (P-EASY PLUS), PAE was associated with reductions in prostate volume, symptoms, detrusor pressure, and the proportion of patients classified as obstructed at follow-up [24]. However, the study lacked a randomized comparator, only a subset completed follow-up urodynamics, and improvement after treatment is not equivalent to proof that future decompensation was prevented.

Taken together, the evidence supports three conclusions: PAE has a true therapeutic effect; it can improve objective obstruction in at least some patients; and it may be acceptable earlier than surgery for selected men. It does not yet support the stronger causal claim that earlier PAE changes the natural history of bladder remodeling.

Comparative Evidence: Prostatic Artery Embolization Versus Transurethral Resection of the Prostate and Holmium Laser Enucleation of the Prostate

Comparisons with TURP expose the central tradeoff. In the randomized trial by Abt et al. [5], short-term symptom improvement after PAE approached that after TURP, but noninferiority was not established and TURP produced substantially greater improvements in Qmax, PVR, prostate volume, and pressure-flow de-obstruction [5]. At two years, symptom and functional improvements remained inferior after PAE, and 21% of patients assigned to PAE had required TURP for an unsatisfactory result [25]. Five-year follow-up confirmed less improvement after PAE in patient-reported and objective outcomes [6].

These findings matter more for a disease-interception claim than for symptom treatment alone. If preservation of bladder function depends on sufficiently reducing outlet resistance, the lesser de-obstructive effect of PAE could limit its preventive value even when symptoms improve. Conversely, the lower number of perioperative adverse events and better preservation of ejaculatory function may make PAE acceptable to a patient who would otherwise decline an operation [5,6,25]. The relevant comparison is therefore not efficacy versus safety in isolation, but whether earlier use of a less complete intervention produces a better lifetime outcome than waiting for a more definitive operation.

Holmium laser enucleation of the prostate (HoLEP) provides another important comparator because it is highly effective across a broad range of prostate sizes. A retrospective comparison found greater one-year improvements in IPSS, quality of life, and tissue-volume reduction after HoLEP than PAE, although the nonrandomized design and unequal group sizes limit inference [26]. A network meta-analysis found no statistically significant difference in several subjective outcomes at one year but greater early Qmax improvement with HoLEP; heterogeneity, indirect comparisons, and limited PAE data reduced certainty [27].

The comparison with HoLEP prevents a false dichotomy between early PAE and delayed TURP. Modern patients may choose among medication, PAE, TURP, HoLEP, and other minimally invasive or surgical options depending on anatomy, prostate size, goals regarding ejaculation, anesthesia risk, access to expertise, and the need for rapid or maximal de-obstruction. A trial of disease interception should therefore justify why PAE is the experimental strategy and should document subsequent crossover to all definitive procedures.

Observational PAE cohorts provide useful estimates of effectiveness but cannot resolve comparative causality. A prospective international study of 478 patients showed sustained symptom improvement through two years; among patients treated for AUR, 65.8% achieved catheter independence by three months and remained catheter-free at two years [28]. A single-center cohort of 1,075 patients reported durable symptom benefit and high short-term catheter-free rates, but attrition at later follow-up and the absence of a concurrent control limit conclusions about natural-history modification [22].

Areas of agreement, controversy, and uncertainty

Several areas of agreement emerge across urologic and interventional evidence. First, PAE improves LUTS and quality of life beyond sham treatment and can improve Qmax, PVR, prostate volume, and selected urodynamic measures [20-24,28]. Second, TURP and HoLEP generally provide more complete objective de-obstruction [5,6,25-27]. Third, PAE usually imposes a lower immediate procedural burden and better preserves ejaculation, although it requires arterial access, iodinated contrast, radiation, and specialized expertise [5-7,22]. Fourth, no existing comparative trial has tested prevention of AUR or preservation of long-term detrusor function as its primary objective.

The principal controversy concerns how much objective de-obstruction is necessary to change long-term bladder outcomes. Symptom relief and physiologic relief are related but not interchangeable. PAE may reduce outlet resistance enough to interrupt remodeling in selected patients, or its lesser effect may only delay a later definitive procedure. Existing trials cannot distinguish these possibilities because they were designed around symptom and flow outcomes rather than longitudinal bladder-function trajectories.

A second controversy is reversibility. Human molecular and morphological studies support partial regression of remodeling after de-obstruction [2,19], and surgical series show that many men with detrusor underactivity improve after outlet procedures [4,29]. Detrusor overactivity may also decline more after surgical relief than during watchful waiting or medical therapy [30]. These observations undermine an overly deterministic point-of-no-return model: advanced dysfunction is not uniformly irreversible. At the same time, recovery remains heterogeneous, so avoiding delayed treatment may still be valuable if a high-risk group can be identified.

A third uncertainty is prediction. Larger prostates, higher PSA, older age, worse symptoms, low flow, elevated residual volume, and retention volume have been associated with progression or TWOC failure [3,8,9,14]. Yet associations derived from groups do not identify an individual therapeutic window, and several markers are influenced by nonobstructive disease. The absence of a validated prediction model is not a reason to abandon the hypothesis; it is a reason to embed biomarker validation within the trial rather than prespecify unsupported clinical thresholds as established care.

Finally, the evidence base is affected by operator dependence, variation in embolic agents and technique, heterogeneous eligibility criteria, inconsistent definitions of clinical success, crossover, and declining long-term follow-up. These limitations are especially important when extrapolating outcomes from experienced centers to wider preventive use.

Risks and limitations of earlier prostatic artery embolization

Earlier PAE could create harm through overtreatment. Many men with mild or moderate LUTS will remain stable or respond to medication; exposing them to an invasive procedure would offer no preventive benefit. Even when major adverse events are uncommon, PAE can cause postembolization symptoms, urinary infection, access-site or vascular complications, nontarget embolization, ischemic injury, transient retention, and contrast-associated risk. Radiation exposure is an additional consideration in a strategy applied earlier to a larger population [7,22].

Technical success and clinical response depend on pelvic arterial anatomy, atherosclerosis, collateral vessels, operator experience, and completeness of embolization. Unilateral or incomplete treatment may reduce durability. PAE also yields no tissue for histopathologic examination, making appropriate evaluation for prostate malignancy important before treatment.

Retreatment has a different meaning in an early-intervention strategy than in treatment of established symptoms. A patient may undergo PAE, resume medication, and later require TURP, HoLEP, or repeat embolization. The cumulative burden, cost, radiation, complications, and effect of sequential procedures must be measured rather than judging the initial recovery period alone [6,22,25].

The proposed strategy also raises implementation concerns. Wider use would require standardized referral criteria, interdisciplinary evaluation, quality assurance, and access to experienced interventional radiologists. Expansion without validated selection could shift practice based on procedural availability rather than patient benefit. These limitations support controlled investigation and argue against present adoption as routine preventive care.

Future directions and proposed clinical trial

A prospective randomized trial is needed to determine whether earlier PAE can alter the clinical course of progressive BPH rather than merely improve established symptoms. The target population should include patients with evidence of progression despite an adequate trial of medical therapy but without prior AUR, catheter dependence, or advanced detrusor failure. For trial purposes, “progression” would require prospectively defined, reproducible evidence of deterioration despite optimized medical therapy, confirmed on serial rather than single measurements. Candidate domains could include change in Qmax, PVR, prostate volume, symptom burden, or structural or urodynamic measures of bladder function. Because validated thresholds identifying impending detrusor decompensation are not currently available, the magnitude, duration, and number of qualifying changes would need to be prespecified in the trial protocol and applied uniformly before enrollment. Patients with neurogenic bladder dysfunction, urethral stricture, bladder malignancy, active urinary infection, bladder calculi requiring treatment, severe renal impairment, or vascular anatomy unsuitable for embolization should be excluded.

Eligible patients could be randomized to continued optimized medical management or PAE in addition to guideline-directed follow-up [7]. A third TURP arm would provide information regarding the relative degree of de-obstruction, but it may be less acceptable to patients who have not yet developed conventional indications for surgery. A pragmatic initial trial comparing PAE with continued medical therapy would more directly address the proposed disease-interception hypothesis.

The primary endpoint should reflect clinical progression rather than symptom improvement alone. A time-to-event composite could include first occurrence of AUR requiring catheterization, recurrent urinary retention, progression to chronic catheter dependence, development of an accepted indication for surgical treatment, or predefined deterioration in bladder emptying. Because individual components differ in clinical importance, each outcome should also be analyzed separately. Secondary endpoints should include changes in IPSS, quality of life, maximum urinary flow rate, post-void residual volume, prostate volume, bladder-wall thickness, renal function, sexual function, medication use, subsequent procedural treatment, and treatment-related adverse events.

Assessment of bladder function is particularly important to the proposed mechanism. Baseline and longitudinal uroflowmetry and post-void residual measurements should be obtained in all participants. A urodynamic substudy could evaluate changes in detrusor pressure, bladder outlet obstruction index, bladder contractility index, compliance, and bladder sensation. Ultrasound or magnetic resonance imaging could be used to assess prostate volume and structural markers of bladder remodeling. These data would help determine whether PAE affects the underlying progression from compensated obstruction to detrusor decompensation [2,13,19].

Follow-up should extend for at least three to five years because AUR, recurrent retention, and bladder decompensation may develop gradually. Randomization should be stratified by age, baseline prostate volume, post-void residual volume, urinary flow rate, symptom severity, and use of combination medical therapy. Prespecified subgroup analyses could then identify the clinical phenotype most likely to benefit from earlier intervention.

The trial would need to address the competing risks of undertreatment and overtreatment. Earlier PAE could reduce progression and preserve bladder function, but it could also expose patients with otherwise stable disease to an unnecessary procedure, contrast administration, radiation, vascular complications, and future retreatment [6,22]. Therefore, the objective should not be to expand PAE indiscriminately, but to determine whether objective markers can identify a subgroup in whom the expected consequences of continued progression exceed the risks of earlier embolization.

Discussion

This review evaluates a disease-interception hypothesis rather than a recommendation to change practice. The biological premise is that sustained bladder outlet obstruction can produce progressive remodeling and that relief before advanced decompensation may preserve function [2,13,17-19]. The clinical premise is that PAE has a lower immediate treatment burden than transurethral surgery and may therefore be acceptable during a stage when patients otherwise continue medication or observation [5,6,20-22]. Both premises are plausible; neither establishes preventive effectiveness. Population-based studies further demonstrate that AUR remains a clinically relevant component of the disease burden associated with BPH [31,32].

The strongest evidence supporting investigation is the convergence of several findings. Medical therapy reduces but does not eliminate progression [8,9]. Symptoms can be discordant with obstruction and bladder function [2,7,12]. PAE has efficacy beyond placebo, outperforms combined medication on several short-term outcomes, and can improve urodynamic obstruction [20,21,23,24]. These observations create clinical equipoise for a trial in a narrowly defined high-risk group.

The strongest argument against premature adoption is equally important. TURP and HoLEP generally produce greater objective de-obstruction, while PAE has inferior durability and greater retreatment in comparative follow-up [5,6,25-27]. No validated combination of PVR, Qmax, prostate size, symptoms, morphology, or urodynamics identifies patients approaching irreversible detrusor failure. Moreover, recovery after surgery remains possible even in patients with detrusor underactivity [4,29]. The hypothesis must therefore avoid presenting AUR as proof that an earlier curative window was necessarily missed.

The proposed high-risk phenotype should therefore be treated as a trial-enrichment concept. Repeated objective deterioration despite verified adherence to appropriate therapy, demonstrable benign prostatic obstruction, progressive gland enlargement, and early bladder changes may be reasonable components. Alternative causes of LUTS or impaired emptying must be excluded. Thresholds should be prespecified and validated rather than selected after outcomes are known.

The trial must test a lifetime tradeoff: whether the lower initial burden of PAE produces fewer episodes of retention, better preserved bladder function, and acceptable cumulative retreatment compared with optimized medical management. Patient-reported outcomes remain important, but they cannot substitute for progression endpoints. Crossover, subsequent operations, medication use, radiation, renal outcomes, sexual function, and health-care utilization should be counted in both groups.

Until such evidence exists, earlier PAE should remain investigational. The value of the hypothesis is to redirect research from short-term symptom comparison toward validated risk prediction, bladder-function preservation, and cumulative treatment burden. A negative trial would be clinically informative by defining the limits of earlier embolization; a positive trial could identify a new role for PAE that is distinct from replacing TURP or HoLEP.

## Conclusions

Progressive bladder outlet obstruction from BPH can be associated with structural and functional bladder changes, although the timing and reversibility of these changes are heterogeneous, particularly among patients with detrusor underactivity or advanced remodeling. Current symptom-directed management is effective for many patients, but symptoms alone may not identify all individuals progressing toward AUR, recurrent retention, or bladder decompensation. Increasing post-void residual volume, declining urinary flow, progressive prostate enlargement, and structural or urodynamic evidence of bladder dysfunction may help define a higher-risk phenotype.

PAE offers meaningful symptom improvement with a lower procedural and sexual adverse-effect burden than transurethral surgery, although TURP provides more complete and durable objective de-obstruction. The available evidence does not establish that early PAE prevents AUR or preserves bladder function. It does, however, provide sufficient biological rationale and clinical equipoise for prospective investigation. A randomized trial focused on disease progression, bladder function, and long-term procedural outcomes is needed to determine whether carefully selected patients benefit from PAE before retention and advanced detrusor dysfunction occur.
